# Consumer-driven strategies towards a resilient and sustainable food system following the COVID-19 pandemic in Australia

**DOI:** 10.1186/s12889-022-13987-z

**Published:** 2022-08-12

**Authors:** Katherine Kent, Fred Gale, Beth Penrose, Stuart Auckland, Elizabeth Lester, Sandra Murray

**Affiliations:** 1 School of Health Sciences, Western Sydney University, Locked Bag 1797, Penrith, NSW 2751 Australia; 2 School of Health Sciences, University of Tasmania, Tasmania, Australia; 3 School of Social Sciences, University of Tasmania, Tasmania, Australia; 4 Tasmanian Institute of Agriculture, University of Tasmania, Tasmania, Australia; 5 Centre for Rural Health, University of Tasmania, Tasmania, Australia; 6 Institute for Social Change, University of Tasmania, Tasmania, Australia

**Keywords:** COVID-19, Food system, Food insecurity, Food access, Community resilience, Sustainability

## Abstract

**Background:**

The COVID-19 pandemic and associated public health restrictions temporarily disrupted food supply chains around the world and changed the way people shopped for food, highlighting issues with food systems resilience and sustainability. The aim of this study was to explore consumer-driven strategies towards a more resilient and sustainable food system in Australia, learning from experiences during the beginning of the COVID-19 pandemic.

**Methods:**

During May–June 2020, a cross-sectional, online survey was conducted in Tasmania, Australia in a non-random sample of adults aged 18 years and over. The survey collected demographic data and posted the open-ended question: “How could Tasmania’s food system be better prepared for a disaster in the future?” Descriptive statistics were used to analyse the demographic data and thematic analysis was employed to analyse the qualitative data.

**Results:**

Survey respondents (*n* = 698) were predominantly female (79%), over 55 years of age (48%), university educated (70%) and living with dependents (45%). Seven key themes were identified: (i) balance food exports with local needs; (ii) strengthen local food systems; (iii) increase consumer awareness of food supply chains; (iv) build collaboration and connection in the food system; (v) embed clear contingency arrangements; (vi) support community capacity building and individual self-sufficiency; and (vii) the food system coped well.

**Conclusions:**

The consumer-driven strategies identified indicate multiple opportunities to increase resilience and sustainability in the food system to avoid future supply disruptions. Our findings indicate that considerable popular support for more resilient, local and sustainable food systems may be emerging from the COVID-19 pandemic.

**Supplementary Information:**

The online version contains supplementary material available at 10.1186/s12889-022-13987-z.

## Background

The global food system, remarkable in its breadth and complexity, comprises a number of smaller food systems with a complex network of actors, covering activities from food production to waste disposal [1, 2]. The global food system is under increasing pressure due to growing population sizes, increasing demand for nutritious foods, biodiversity impacts of farming, and climatic pressures [3]. With regard to the latter, it has been estimated that the global food system generates about one-third of total annual greenhouse gas emissions, with food production, land use, and food distribution mainly responsible [4]. There is also clear evidence of significant issues with social equity and food access inequality, with over 800 million people food insecure at one extreme; while over one billion people suffer from over nutrition and obesity [5].

Australia, an agricultural nation, is deeply enmeshed in a ‘world food economy’ [6] with 70% of Australia’s produce being exported internationally [7]. While this makes Australia ‘food secure’ in one sense, there are vulnerabilities in regional and local food supply chains that warrant closer attention. Australia’s food supply chains are predominantly composed of complex business networks that are vulnerable to geopolitical, environmental, economic and societal shocks. In previous years, the resilience of Australia's food supply chain following disasters has been questioned, with the fragility of both long and short supply chains queried [8]. The COVID-19 pandemic demonstrated vulnerabilities in the Australian food system, which were echoed within food systems across the world, from global down to the local levels [9]. However, the pandemic also presented opportunities to reflect on how to make food systems more resilient [10] and sustainable [11]. Food system resilience has been defined as having “the capacity that ensures stressors and shocks do not have long-lasting adverse consequences” [12]. Resilient food systems contribute to food security [9] and, ultimately, to sustainable food systems [13, 14].

Internationally, the COVID-19 pandemic impacted food systems through shifts in consumer demand and disruptions in suppliers’ capacity to produce and distribute food [15–17]. Primarily, the pandemic’s associated public health restrictions at national and international levels disrupted established food supply and demand arrangements, even within seemingly well-established supply chains [18]. This affected all four elements of food security: the availability, stability, access, and utilization of food [19]. In Australia and internationally, vulnerabilities with long and complex food supply chains have been brought to light. While long supply chains can provide inexpensive food year round, the unexpected surges in demand for food at the beginning of the pandemic saw supermarket shelves emptying and suppliers scrambling to restock them. Concerned by possible shortages, consumers adjusted how they acquired and consumed food while concurrently coping with a range of restrictions on transportation and free movement within the community [20]. The dramatic nature of these changes, and their impact on food insecurity [21], underscores the urgent need to better understand such shock events and inform preparations for food systems to cope with foreseeable future disruptions [22].

In the midst of the disruption caused by the pandemic, an important opportunity opened up to reflect on current arrangements and consider what is required to build more resilient and sustainable food systems [13]. While many voices are worth listening to, an important one is that of food consumers who, through the economic-focussed discourse of ‘consumer sovereignty’, are often claimed to influence the food supply they demand. Indeed, recent research has identified that Australia must prioritise engaging consumers in developing healthy, safe and sustainable food systems [23]. However, the current industry focus on high profits and margins obtained through exports can easily generate food supply chains that disadvantage local end-users [24]. We examine this issue in the Australian context, which in addition to the pandemic, has recently experienced localised food supply issues following natural disasters such as bushfires and floods [25]. While the COVID-19 pandemic has impacted communities across the globe in different ways, Tasmania, Australia is a rural and regional, low-socio-economic region located in a rich, developed, first world country, which has lessons for other similarly situated regions in countries like Britain, Canada and the United States. Case studies can provide the base for subsequent comparative studies of the impact of COVID-19 on food systems and food insecurity. Therefore, the aim of this study is to better understand the food consumers’ experience of the COVID-19 pandemic and their views on what a resilient and sustainable food system might look like, based on a detailed case in Tasmania, Australia.

## Methods

### Study setting

Tasmania/lutruwita is Australia’s only island state, located 240 km southeast of the Australian continent across a body of water known as the Bass Strait. Its population of about 515,000 people is the most regional and dispersed of any Australian state, with 57% living outside densely populated areas [26], and almost five percent identifying as Indigenous. Since the downturn of the forestry industry [27], Tasmania’s economy has experienced a revitalisation in Agri-tourism, enhancing its reputation for producing some of the best quality food produce in Australia and developing a strong food-based visitor economy.

The Tasmanian government’s response to this economic opportunity has seen the launch of export-oriented agribusiness and agri-tourism strategies based on economic targets [28]. However, recent concerns regarding unsustainable food production, including for farmed salmon have been published [29]. Despite this, Tasmania maintains a “clean and green” brand with a thriving organic and regenerative agriculture industries and a recently extended Genetic Modification (GM) moratorium to 2029. The food environment is dominated by two major supermarket chains located in major population centers [30]. Fruit and vegetable shops and local independent supermarkets and specialty stores are often located in regional towns and outer suburbs. General and convenience stores, predominantly owned by local families, are located in more regional, rural and remote communities. A small number of farmers’ markets sell locally grown produce at weekends [31]. Independent supermarkets in Tasmania specialize in sourcing local food, however experience difficulties in competing on price with the major supermarkets who import food to the island from the Australian mainland. One major supermarket owns the only grocery distributor in Tasmania, which means the second major supermarket imports all its goods from the mainland, creating a supply chains that rely heavily on interstate and international transport and which are vulnerable to external shocks.

### Food system disruptions at the beginning of the COVID-19 pandemic

On 17 March 2020, Tasmania declared a public health emergency in response to the COVID-19 pandemic, which gave stronger powers and sanctions to the director of Public Health [32]. Thirteen days later, a strict lockdown for four weeks was imposed. In response to a COVID outbreak in the North-west and West of Tasmania, further lockdowns were imposed forcing the closure of non-essential businesses and travel [33]. Travel restrictions on interstate travellers, especially from Victoria and New South Wales, were also imposed. As a result of these measures, Tasmania avoided having any large community outbreaks of COVID-19 and restrictions began to be eased on 9 June 2020 [34]. While the island’s borders remained closed to visitors from pandemic ‘hot spots’, Tasmania was effectively COVID-free (without any community transmission) from 6 May 2020 until December 2021.

At the beginning of the pandemic, there was a significant surge in demand for food as people bought large quantities of food in anticipation of being at home for long periods of time [35]. With widespread panic buying, supermarkets were forced to restrict the sale of certain items such as rice, tinned goods, and minced beef, potentially fuelling the impression of a shortage [36]. Some hospitality businesses were forced to close due to social distancing restrictions; others adapted their business model to offer takeaway or home delivery [36]. The way in which consumers shopped in supermarkets also changed as individuals were encouraged to reduce the number of shopping trips taken to avoid the risk of infection, have only one member of the household shopping for food and alter the timing of the trip to avoid peak times [37]. There was also a significant increase in food deliveries and ‘click and collect’ shopping [37].

### Data collection

The study was conducted in collaboration with The Tasmania Project, a University of Tasmania initiative established by the Institute for Social Change to understand how residents are experiencing and adjusting to the social, political, and economic responses to the COVID-19 pandemic. Participants were recruited using convenience sampling methods, with the survey link promoted through social media, traditional media interviews and through community organizations. Email invitations were distributed to potential participants who had signed up for updates related to previous research undertaken by the Tasmania Project. From 25th May to 7th June 2020, The Tasmania Project’s Food Survey invited survey responses from a non-random sample of Tasmanian residents aged 18 and over about how food access and supply had changed in during the COVID-19 pandemic. This included their opinion about how the food system could be future-proofed against further threats. Participants used a link to enter the survey and were asked to read the participant information sheet. Participants gave informed consent and were also screened for eligibility to ensure they were aged 18 years and over and living in Tasmania. Eligible, consenting participants completed an online, self-administered survey through SurveyMonkey.

This study presents the results of the open-ended survey question which asked respondents to describe “How could Tasmania’s food system be better prepared for a disaster in the future?” Nine quantitative demographic questions were also collected including age, gender, citizenship status, employment status, household composition, their local government area, whether they had a disability, identified as an Indigenous person (Aboriginal and/or Torres Strait Islander), and their highest level of education. The study was conducted in accordance with the Declaration of Helsinki, and the protocol was approved by the University of Tasmania’s Social Sciences Human Research Ethics Committee (Ethics Project ID: 20,587).

### Data analysis

Demographic characteristics were exported into SPSS and analysed using descriptive statistics. Chi-square test was used to compare differences in the demographic characteristics between participants who responded to the qualitative question and the entire sample. Qualitative responses from the consumer survey were exported from SurveyMonkey into an Excel spreadsheet. A six-step method to thematic analysis strategy was employed [38] where ‘thematic analysis involves searching across a data set to find repeated patterns of meaning’ [38]. Two researchers (KK, SM) led the analysis. KK is a female public health nutritionist (PhD) employed as a lecturer, with 5 years of qualitative research experience. SM is a female dietitian (RD and PhD candidate) with over 10 years of experience as a researcher. Separately, KK and SM reviewed the free text responses to become familiar with the content. Notes were taken during the analysis to stimulate reflection at each stage by both SM and KK. Individually, using an inductive approach, the free text responses were searched for recurrent themes that were derived from the data. Codes were iteratively developed by generating succinct labels for important features of the data. These labels were collated to generate initial themes. After bringing these codes together, SM and KK discussed the codes length and together they edited the initial themes to generate a preliminary thematic framework. The thematic framework was discussed, reviewed and refined between the two reviewers (SM KK), and disagreements were resolved through a process of consensus. The process was iterative and utilised notes taken throughout the analysis to stimulate reflection. The thematic framework went through several iterations, following discussions and reflections with co-authors and the final themes were thoroughly checked by the other team members. In consultation with the wider project team (FG – male, professor, political economist PhD, SA – male, Masters, lecturer in rural health, and BP – female, PhD, agricultural scientist), and a description and an informative name was developed for each theme before writing a narrative synthesis of the results [29]. Criteria in the COREQ, a 32-item checklist was utilised to report important aspects of the methodology [39] (Supplementary File 1).

It is acknowledged that the research team’s prior knowledge and experiences in working in food security, nutrition and food systems research inevitably affected the analysis and therefore reflexive thematic analysis was important in ensuring the research was rigorous and credible [40]. The research team come from diverse backgrounds (nutrition (KK, SM), rural health (SA), agriculture (BP) and political economy (FG)) providing a balanced framework for the analysis which may mitigate the tendency of bias to skew the results of our analysis.

## Results

Of the 1,170 respondents who entered any valid data to The Tasmania Project’s Food Survey, 698 respondents (60%) entered a qualitative response to the open-ended question. Table 1 shows the demographics of participants who provided a qualitative response and relevant demographic characteristics (demographics of the entire sample have been previously reported [21]). All qualitative responses were utilised in the analyses regardless of whether data for demographic characteristics was received, resulting in smaller numbers for some characteristics (Table 1). There were no significant differences observed between the demographic characteristics of the sample that provided a qualitative response compared to the entire sample of the Tasmania Project.

**Table 1:** Demographic characteristics of the study sample (*n* = 698)

Characteristic	Categories	n	%
Age (*n* = 668)	18–25	14	2.1
	26–35	66	9.9
	36–45	109	16.3
	46–55	156	23.4
	56–65	186	27.8
	65 +	137	20.5
Gender (*n* = 684)	Female	542	79.2
	Male	139	20.3
	Other/prefer not to say	3	0.4
Region (*n* = 684)	South	407	59.5
	North	165	24.1
	North West and West	112	16.4
Highest Level of Education (*n* = 682)	University	477	69.9
	Diploma/TAFE	131	19.2
	High School	74	10.9
Disability Status (*n* = 683)	Disability	167	24.5
	No Disability	516	75.5
Aboriginal and/or Torres Strait Islander Status (*n* = 681)	Yes	14	2.1
	No	667	97.9
Citizenship Status (*n* = 683)	Born in Australia	531	77.7
	Born overseas, Australian citizen	123	18.0
	Born overseas, permanent resident	20	2.9
	Born overseas, temporary resident	9	1.3
Household Structure (*n* = 681)	Couple family with dependents	308	45.2
	Couple family without dependents	184	27.0
	Single parent family with dependents	36	5.3
	Living alone	124	18.2
	Other (e.g. group/share/living with parents)	29	4.3

**Fig. 1:**
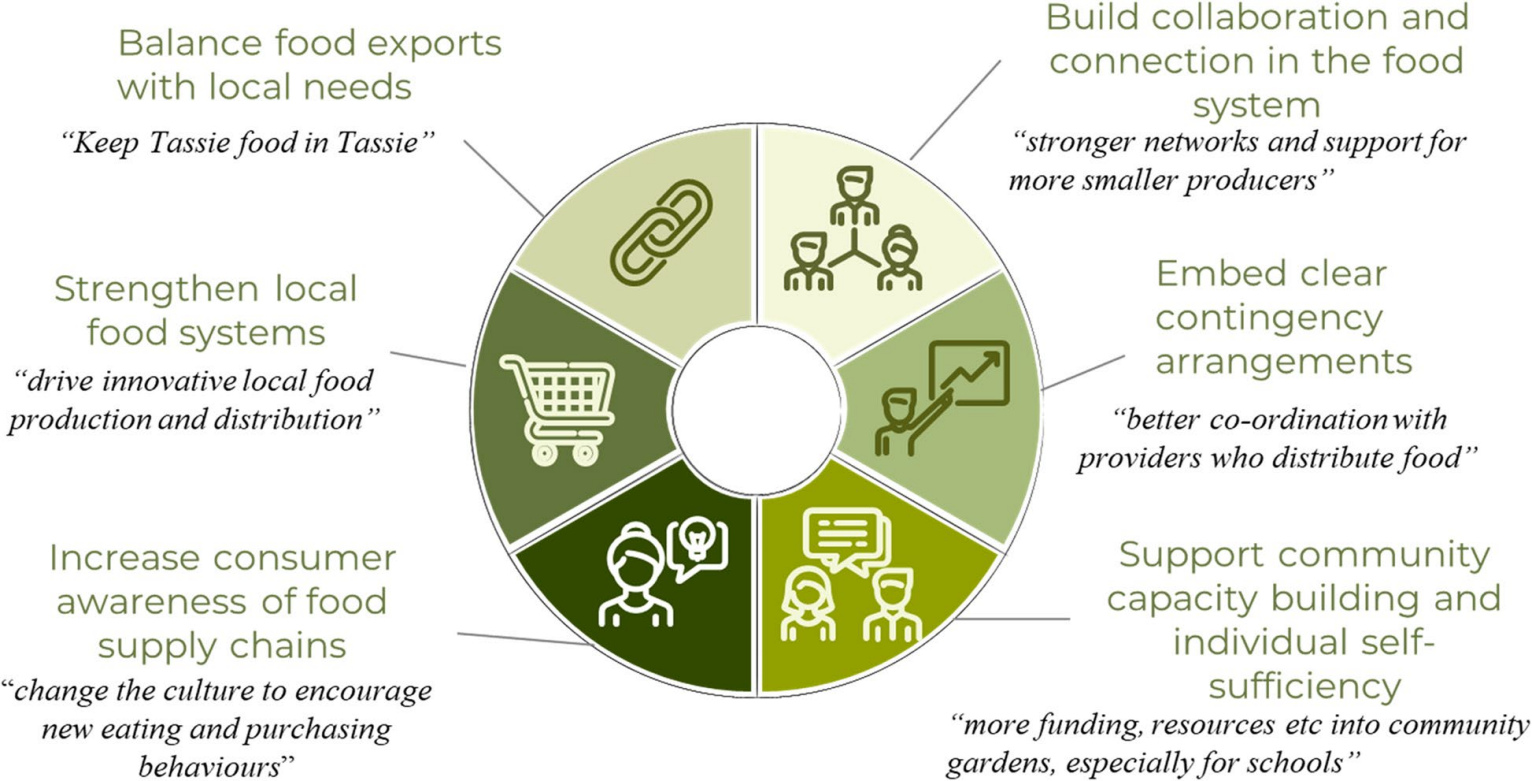
Six of the seven key themes identified in the thematic analysis indicate key action areas to build a resilient and sustainable food system

Seven key themes were identified following the thematic analysis: (i) balance food exports with local needs; (ii) strengthen local food systems; (iii) increase consumer awareness of food supply chains; (iv) build collaboration and connection in the food system; (v) embed clear contingency arrangements; (vi) support community capacity building and individual self-sufficiency; and (vii) the food system coped well. Six of these themes indicate key action areas to build a resilient and sustainable food system (Fig. 1).

### Balance food exports with local needs

Participants were strongly of the opinion that the state could grow sufficient food to meet the needs of its population and frequently referenced the tension between balancing food exports against local needs. Consumers expressed a desire for local food producers to prioritise and meet the needs of the local community and *“Keep Tassie food in Tassie”*, before growing produce for national and international export. The possibility of self-sufficiency at a state-level was commonly described: *“Tasmania should be completely self-sufficient, only importing what is out of season or cannot be grown here (at high taxed rate) and only exporting what is excess to the State needs”* (Female, 39 years). The potential of a self-sufficient strategy to keep businesses functioning in future disasters and pandemics was also discussed. *“Businesses need to have the right balance of exporting their produce and supplying locally. They won’t close their doors if they aren’t relying too heavily on the export market”* (Female, 50 years). The noted tension over food exports emerged strongly when consumers discussed food tourism: *“Don’t pretend that the economy depends on tourism”* (Male, 44 years). Respondents wanted clear strategies to *“address the gap between Tasmanian producers and Tasmanian consumers—most of the food we produce is exported, much of the food we eat is imported.”* (Female, 35 years).

Whilst Tasmania has an outstanding reputation for producing premium food and wine, consumers reported these foods did not match local needs, and were out of reach for most residents: “*Currently, it seems like buying locally is restricted to the more wealthy or to people like myself ‘as a treat’"* (Male, 30 years). Similarly, respondents identified that the affordability of locally-grown food could be improved for some: *“Focus on all demographics”* (Female, 55 years*)* and *“produce more affordable food in Tasmania instead of artisan products only”* (Female, Age not disclosed).

The positive implications for both consumers and food producers of relying on local market produce was discussed: *“Producers who provide for local consumers as their primary source of income are more resilient to disaster”* (Female, 46 years). Contrasting views were presented on whether the food system could *“Continue to diversify”* (Female, 43 years), as a strategy to ensure self-sufficiency, and whether stockpiling foods grown outside the state was a possibility: *“State (government) might 'encourage' strategic stockpiles in state due to our isolation by sea from mainland logistic routes”* (Male, Age not disclosed).

### Strengthen local food systems

Consumers commonly reported that actions could be taken to shorten food supply chains to protect against future shocks: “*It doesn't feel secure to rely on 2–3 private owned supermarket chains*” (Male, 44 years). Consumers also expressed a desire to be *“less reliant on Bass Strait imports”* (Female, 65 years) [the Bass Strait is the body of water separating the island of Tasmania from mainland Australia]. Sometimes, consumers identified the potential for further investment in small scale farming and distribution networks as a key strategy to improve resilience: *“there is an opportunity for local government/not-for-profit to act as an enabler to drive innovative local food production and distribution—including availability to all communities at reasonable price”* (Female, Age not disclosed).

Strategies to support a consumer’s ability to buy locally-grown produce were discussed and included greater marketing and clearer labelling of locally-grown food: *“there needs to be clear labelling so we know a foods provenance”* (Female, 73 years)*.* Greater support for locally owned and operated growers and manufacturers was also discussed. Additionally, some consumers stated that further protections against selling prime agricultural land for foreign investment could protect the local food supply in the future: *“be more conscious of issue of foreign ownership of agricultural land”* (Female, 73 years).

### Increase consumer awareness of food supply chains

Consumers appeared to be aware of the structure of the current food system. Major supermarkets were seen as important food retailers, but a common view was that these outlets needed to adapt to support shorter supply chains and prioritise locally-grown produce. Consumers suggested that supermarkets could do this either *“of their own volition”* or be *“forced to pay local producers’ reasonable prices and stock a wide range of local produce”* (Male, 70 years)*.* It was claimed these changes would not only support consumer values but also provide farmers with the *“confidence to supply local markets [in the] long term”* (Female 65 years). Some consumers also suggested that there was opportunity to improve the local food culture to “*Take pride in and buy Tasmanian produce”* (Female, 44 years). The view was expressed that if all residents prioritized locally grown purchases, the Island could protect itself against future shocks to the food system: *“educate people and change the culture to encourage new eating and purchasing behaviours that prioritise consumption of seasonal and local produce”* (Male, 38 years).

### Build collaboration and connection in the food system

Respondents suggested there are opportunities to promote stronger connections between local food producers through initiatives such as small grower networks which could provide *“stronger networks and support for more smaller producers”* (Male, 50 years). These networks were considered by some to contribute to contingency planning and providing support and connection for a more responsive food system in future disaster-type situations: *“Producers need to have systems in place which would allow them to quickly change the way in which they supply their customers. e.g. online service, home delivery”* (Female, 71 years). At the beginning of the pandemic, food suppliers and producers were forced to establish new ways of getting food to the people, which respondents valued. This included online platforms which *“allowed more direct connection between groups of growers and consumers”* (Male, 45 years).

### Embed clear contingency arrangements

Consumers expressed a desire for the government and businesses to develop and implement food-related disaster preparedness plans: *“there should be detailed and improved logistics and supply chain systems for pandemics and disasters, implemented even prior to a pandemic or state of emergency being announced to prevent supply shortages”* (Female, 50 years)*.* Some consumers suggested that plans would include both immediate rationing and longer-term plans. Consumers identified that there was a lack of a coordinated response, which impacted food supply chains and *“better co-ordination with providers who distribute the food”* (Female 49 years) would be required in the future. The need for *“stronger political control”* (Male 44 years) in future pandemics or disasters was emphasised, as industry-implemented restrictions developed to minimise the effects of panic-buying and hoarding were considered too little and too late. Consumers thought that the *“rationing imposed by shops is ineffective and unfair”* (Female 72 years), reporting they disproportionately affected some households*: “Some larger families I spoke to struggled to get enough food to feed their families”* (Female, age not disclosed).

In addition to reducing hoarding, some consumers suggested developing contingency plans for redistributing locally grown food through a network or coalition of stakeholders. Such plans would support local producers and develop “*a better understanding of the food system by connecting foodies at a regional scale *via* a dedicated food-systems group”* (Male 41 years). In the longer-term, ongoing *“counter-disaster discussion meetings”* (Female, 80 years) with diverse food industry stakeholders were suggested. Lastly, consumers sometimes discussed the reliance on overseas workers to support primary producers as a weakness or threat to food security during periods of disaster or pandemics: *“the heavy reliance on overseas workers to harvest produce is one of the biggest weaknesses exposed by COVID19”* (51 years, gender not disclosed).

### Support community capacity building and individual self-sufficiency

Consumers suggested there were opportunities for governments to invest in community capacity building and to promote self-sufficiency at an individual level. It was understood that such support was not simply a disaster response and could assist a transition to a ‘new normal’. Suggested initiatives included support for individuals to understand how to grow food, learning permaculture, and composting to be more self-sufficient and *“grow their own staple vegetables and fruit”* (Female 46 years). Additionally, nutrition education was suggested including healthy cooking and seasonality: *“If more people were aware of the seasonality of produce, they may be less expectant to have things available all year round and not freak out when suddenly one particular item isn't available”* (Female 28 years). Lastly, support for community initiatives to support food literacy and self-sufficiency was suggested as a priority for some: *“Put more funding, resources *etc. *into community gardens, especially for schools”* (Female 51 years).

### The food system coped well

Most respondents reported being affected by changes to the food system during the pandemic: *“we weren't sufficiently prepared”* (Female 50 years)*.* However, some respondents reported they were resilient to supply chain challenges through being flexible by utilizing the food that available, even if different to their usual purchases, which may relate to high food literacy in some participants: *“there was still plenty of food, just not necessarily everything we are used to”* (Female 53 years)*.* Rarely, respondents suggested the food system was robust throughout the beginning of the pandemic: *“I think our supply chain is efficient and well organised”* (Female, 69 years).

## Discussion

This cross-sectional study conducted during strict social distancing restrictions at the beginning of the COVID-19 pandemic assessed consumer perceptions of possible strategies to build a more resilient and sustainable food system. Focused on Tasmania, Australia, our results provide an understanding of how consumers perceived the food system considering the issues they encountered during the beginning of the pandemic, in addition to offering potential solutions generated by consumers that could be further explored and evaluated by other food system-stakeholders and policymakers as strategies to increase future food system resilience and sustainability. Short and longer-term strategies spanning individual, food industry, logistics and policy levels were identified by consumers with a focus on addressing concerns of local versus exported food, cheap versus luxury food, supermarkets versus alternative food outlets, increasing supply chain transparency, and focussing on disaster preparedness.

### Local versus exported food

A major finding of our study is the perceived tension that exists between consumers and food producers over whether to prioritise growing food for export or domestic markets. The vast majority (89%) of respondents to the Tasmania Project’s Food Survey reported that they valued locally grown produce, and more than half (54%) agreed that locally-grown produce had become ‘more important’ than before the pandemic [41]. However, it is currently unclear how much food bought and consumed by Tasmanians is locally grown, undermining our understanding of the feasibility of local food systems for the economic viability of the food system. Reconciling tensions between domestic and export production is unlikely to occur in the short term, given that 78% of the food grown in Tasmania is exported to mainland Australia or overseas [42], putting producers in a dominant position over consumers. However, the implications of reducing food exports in favour of a local market are difficult to balance, given that the seasonality of locally-grown food production make export of food practicable and profitable. Indeed, in pure financial terms, the Tasmanian agri-food sector contributed AUD$3.95 billion in retail and food services sales, AUD$3.05 billion in interstate sales and AUD$0.77 billion in sales of overseas exports in 2018–19 [43], and the Tasmanian govenerment has a target of annual farm value of AUD$10 B by 2050 [44]. From the perspective of government and industry, there would be little practicality in moving from an export-heavy to local-only food market. However, this study supports the findings of previous studies [45, 46] that suggest that making local produce more available in conjunction with better consumer education via improved provenance labelling and seasonality awareness could satisfy desire to purchase local food without compromising exports.

It has been reported that food producers are concerned that a localised food system based on local procurement may not be possible as it is perceived that the local market is not profitable enough [42]. However, during the COVID-19 pandemic, small cracks in the export narrative appeared, reflected in that 43% of respondents to The Tasmania Project’s Food Survey reported buying either ‘a lot more’ or ‘somewhat more’ locally grown produce, and a further 49% reported buying ‘about the same amount’ [41]. The claimed change in consumer behaviour may relate to the fact that interstate supply chains were disrupted, and this change may have been forced. However, it might have reflected how local food producers were able to innovate quickly to meet consumer demand [20], demonstrating a degree of supply-chain resilience, signalling that opportunities exist for the growth of shorter, more local, supply chains. Some businesses were able to quickly innovate by employing digital technologies and platforms for producers to sell directly to consumers; restaurants switching to providing take-out and home delivery; and farmers markets converting from open-air to box-based supply schemes [37]. Further support for local entrepreneurship and innovation, including social enterprise within this sector, may strengthen the local market for locally-grown foods.

In our study, consumers perceived those businesses who focussed on local needs were better off during the COVID-19 pandemic lockdowns. This approach contrasts strategies of building food systems resilience following the COVID-19 pandemic in international literature, whereby it has been suggested that export and trade of food “*must be uninterrupted and even facilitated*” through international cooperation [47]. Replacing imports with domestic production may be a high-cost option of maintaining the food system in the longer-term [48], so the Government and producers might consider the balance between strengthening institutions that govern international trade or reversing the impacts of globalisation on their food systems [49]. Continued support by consumers for locally grown produce would assist with strengthening the local market, especially if food exports and international markets continue to be disrupted by pandemics and natural disasters.

### Cheap versus luxury food

Prior to the COVID-19 pandemic, the state was well connected to markets via sea and air, enabling ‘luxury’ foods to flow outwards and ‘cheap’ food, inwards. However, the pandemic interfered with these established trade relationships, at least in the short term, due to unpredictable travel restrictions and quarantine regulations within Australia and in major trading partner countries [47]. Sectors impacted included seafood, red meat, and wine, which are among Tasmania’s biggest food exports. One paradoxical effect of this was that outward-bound luxury food items, including crayfish typically out of reach for many Tasmanians, were redirected for sale locally at heavily discounted prices [50]. This both benefitted consumers and kept many small businesses afloat [51], indicating the strong local support of consumers for Tasmanian produce. Consumers in our study suggested that food producers could continue this supply of local affordable food while also diversifying supply. As Tasmanian previous research has identified that the most substantial barrier to consuming locally-grown produce is high price and limited seasonal availability, the identified problems are not unique to the COVID-19 pandemic [46]. A challenge for food producers and consumers in the future is to work out a way to overcome these tensions between price, variety and locality [52]. Conceivably, however, the steadily rising background demand for locally grown food [53] coupled with heightened awareness and pandemic-induced concerns among consumers over the operation of conventional, industrialized food systems could contribute to the development of a stronger local market.

### Supermarkets versus alternative food outlets

In our study, consumers reported being somewhat concerned about the reliance on major supermarkets within a globalised food system, and perceived that re-localisation of the food supply chain and the development of alternative agri-food networks would be central to building resilience against future disasters [52]. As elsewhere, consumers in our study tended to have very positive associations with local and small-scale farming [54]; however, similar to work prior to the pandemic, they reported difficulty identifying locally-grown produce, especially when sold through supermarket chains, due to a lack of clear provenance labelling [46].

Consumers in our study appeared to be aware of trade-offs concerning these competing preferences. During the beginning of the COVID-19 pandemic, most consumers identified that they had sought locally grown foods from independent supermarkets (75%) *and* major supermarkets (65%), indicating that even for consumers who would like strong local food systems, trade-offs occur between wider concerns of the food system and the pragmatics of shopping for food [53]. Consumers in our study argued that supermarkets could further prioritize locally-grown produce for sale. However, supermarket retailers are not perceived as advocating for and supporting shorter food-supply chains via local procurement strategies. A finding of our study is that supermarkets have a clear opportunity to capitalize on the strong local-food movement by championing “buy local” campaigns. However, the efficacy of any such campaign would depend on unambiguous and agreed multi-stakeholder understandings of what ‘local’ means as otherwise there would be many options for ‘local-washing’, an equivalent to the more common ‘green-washing’ that occurs with environmental and sustainability reporting [55].

### Supply chain transparency

Generally, consumers are increasingly aware of conventional food production processes and are concerned about their food choices [56]. Results from our study suggest that respondents were somewhat knowledgeable about the Australian food system, and some were aware of challenges faced by the industry. However, the complexity and anonymity of food supply chains is still increasing and consumers in our study desired more transparency in the food production process in order to be empowered to make appropriate food choices that build resilience in local food systems [57]. This finding aligns with a study of Australian consumer perceptions of the food system that showed 93% of respondents believed consumer’s lack an awareness and understanding of the food system, but there is growing public and political awareness and support for healthy food environment [23].

Consumers in our study identified that developing strong networks for advice, information distribution and sharing between producers and food system stakeholders—that is greater transparency—would be a strategy to build resilience and sustainability, which is supported by literature [47]. Strong networks of “foodies”–those individuals and groups promoting more local, healthy, seasonal, resilient and sustainable food systems—would facilitate supply chain innovation and contribute to achieving mutually beneficial goals [58]. Previous research shows that strong regional networks with a shared strategic agenda are characterised by greater transparency leading to ‘food democracy’ and improved equity and access [52]. When markets are disrupted, such relationships enable local producers to leverage community networks to find necessary inputs, including labour, and to escalate word‐of‐mouth and social media promotion [20].

### Disaster preparedness

A final major finding in our study was the perception that stronger contingency arrangements at a government and business level would be required to support access to food during future disasters. Contingency plans and mitigation strategies must allow a more rapid response to extreme events and transform the food sector by making it more resilient [19]. Targeted policies would be required to ensure stability of supply, physical access to shops and markets, and economic access to healthy, nutritious foods [47]. Consumers in our study perceived that the restrictions on panic buying at a business level were ineffective and inequitable, and therefore governments must develop strategies to control a quick response, rather than leaving it up to businesses with vested interests in selling food. Representative, multi-stakeholder crisis committees could be established to ensure adequate and full implementation of regionally appropriate strategies [59], and could assist in developing plans for handling local supplier and transport disruptions and continue the innovative work on service models that reduce consumer contact. Strategies that were successful at maintaining the food system during the pandemic, including new online food sales platforms could continue to be supported going forward. At the national level, a national food policy could encourage innovation and coordination between national, state, and local government levels to support food system systems that deliver healthy food across the population.

Some consumers in our study suggested that disaster preparedness might be enhanced if individuals and communities were more easily able to produce their own food, which would build resilience and improve food security [59, 60]. Positively, it has been reported that over a third of respondents started growing more of their own food during the COVID-19 pandemic [61]. Interestingly, 40% of respondents who were food insecure were also growing more of their own food during COVID-19. Many new gardeners may need further help to develop their knowledge and skills to maintain higher levels self-sufficiency in the future. To facilitate this, further investment in education and training programmes is required as a state and national priority [60]. Programs that develop food literacy and food systems thinking could be useful in building food system resilience and may allow the Tasmanian community to respond to the impacts of pandemics in the future.

Lastly, while not a major finding, we note that a minority of respondents in our study suggested that while there some issues, overall, they considered the food supply chains to be resilient enough during the beginning of the COVID-19 pandemic because they did not run out of food, even if they needed to eat different foods than usual. Indeed, shortly after the survey was conducted, grocery store shelves were replenished as consumers reduced the volume of food purchases after initial stockpiling. Given the convenient nature of our study sample, this divergent view should be explored in further detail by future research to determine the extent to which food supply issues impacted Tasmanian households, and the extent to which resilience is related to an individual’s food literacy. Our previously published work shows there was a disproportionate impact on access to food and the availability of food for food insecure households during the pandemic [21]. Therefore, while some respondents to our survey perceived that they were not substantially impacted by changes to the food system, some groups of vulnerable respondents [21] may have been under-represented in our study.

### Strengths and limitations

The strengths of this study include a strong response rate for the open-ended question and the generation of rich and meaningful data that was amenable to thematic analysis. The approach enabled insights to be gained into how people interpreted the challenges of feeding themselves and their families during the initial stages of the COVID-19 outbreak—that is, to the *meanings* they attached to food, food insecurity, local food, food exports, and the Tasmanian food system. The limitations include that a higher proportion of responses were from female, older, and more highly educated participants relative to the Tasmanian population. For example, our sample contained 79% female respondents compared with the demographic profile of the Tasmanian population (51.1% female), which may be explained by the nature of the survey and that women reportedly manage most household meals in Australia. Additionally, our respondents were overall very highly educated, with 69% having a university education, compared with 16% of all Tasmanians that have tertiary qualifications. This limits generalizability of our study findings to the experiences of other demographic subgroups in Tasmania such as males and people from lower education or socioeconomic backgrounds. The results may also not be generalisable to other regions across Australia given the difference in public health restrictions and differences in food policy at the time of the survey. As such, inferences about our study should not be drawn beyond the qualitative sample. Collecting open-ended responses to the questions may have limited respondents to feedback based on the immediate information they are accessing. It is conceivable that our respondents may not have understood the question or had little knowledge about potential solutions to the issue, which may have limited our responses. Such surveys are usefully supplemented by more deliberative approaches to understanding individual’s preferences such as Deliberative Valuation and Q-Methodology [62]. It is hoped that follow up studies may be able to incorporate these additional approaches, time and resources permitting.

## Conclusion and recommendations

This study captured consumers’ views on the opportunities to build resilience in the food system during a time when it was disrupted by the COVID-19 pandemic in Tasmania, Australia. Our findings provide a consumer voice to the food supply impacts during COVID-19 pandemic and identify potential strategies from a consumer perspective on how the food system could be designed to deal with those impacts into the future. Consumers in our study suggested several regional development strategies that may warrant consideration by policymakers who are looking to understand consumer perspectives when designing solutions for a more resilient food system. In particular consumers proposed strategies that would (i) better balance food exports against local consumer needs, (ii) strengthen local food systems, (iii) build strong regional food networks, (iv) support disaster preparedness efforts, and (v) grow self-sufficiency at a community level. Further research into the drivers of adverse impacts of the current food system and determinants of food resilience is warranted. Greater investment in community capacity building could be considered to support consumers to develop food literacy skills and establish resources at the community level. Future strategies are also likely to require closer collaboration between the agencies responsible for implementing policies around food systems and disaster/emergency management. Further research with other food system stakeholders is also required to confirm the current findings and generate a more comprehensive understanding of the steps and actions required to build resilience into food system in Australia and elsewhere.

## Supplementary Information

**Additional file 1. **COREQ (COnsolidated criteria for REporting Qualitative research) Checklist.

## Data Availability

The datasets generated and/or analysed during the current study are not publicly available due ongoing data analysis but are available from the corresponding author on reasonable request.
